# Dynamic regulation of EZH2 from HPSc to hepatocyte-like cell fate

**DOI:** 10.1371/journal.pone.0186884

**Published:** 2017-11-01

**Authors:** Mariaelena Pistoni, Nicky Helsen, Jolien Vanhove, Ruben Boon, Zhuofei Xu, Laura Ordovas, Catherine M. Verfaillie

**Affiliations:** KU Leuven—Department Development and Regeneration, Stem Cell Institute (SCIL), Leuven, Belgium; Ospedale Pediatrico Bambino Gesu, ITALY

## Abstract

Currently, drug metabolization and toxicity studies rely on the use of primary human hepatocytes and hepatoma cell lines, which both have conceivable limitations. Human pluripotent stem cell (hPSC)—derived hepatocyte-like cells (HLCs) are an alternative and valuable source of hepatocytes that can overcome these limitations. *EZH2* (*enhancer of zeste homolog 2*), a transcriptional repressor of the polycomb repressive complex 2 (PRC2), may play an important role in hepatocyte development, but its role during *in vitro* hPSC-HLC differentiation has not yet been assessed. We here demonstrate dynamic regulation of EZH2 during hepatic differentiation of hPSC. To enhance EZH2 expression, we inducibly overexpressed EZH2 between d0 and d8, demonstrating a significant improvement in definitive endoderm formation, and improved generation of HLCs. Despite induction of EZH2 overexpression until d8, *EZH2* transcript and protein levels decreased from d4 onwards, which might be caused by expression of microRNAs predicted to inhibit *EZH2* expression. In conclusion, our studies demonstrate that EZH2 plays a role in endoderm formation and hepatocyte differentiation, but its expression is tightly post-transcriptionally regulated during this process.

## Introduction

Currently, primary human hepatocytes (PHHs) are the gold standard for *in vitro* drug toxicity and metabolization studies. Use of PHHs is however limited due to scarcity of donors, high inter-donor variability and rapid *in vitro* dedifferentiation [1]. Human pluripotent stem cells (hPSCs) have the capacity to differentiate into the three somatic germ layers and all cell types of the body, and are an alternative and renewable source of hepatocytes that could be used for drug toxicity and metabolization studies. hPSC-derived hepatocytes have many advantages over primary hepatocytes and hepatocellular carcinoma cell lines, as they could provide an unlimited supply of hepatocytes from a single donor, limiting inter-donor variability; as well as create cells from a diverse number of patients to study mechanisms underlying drug-induced liver injury (DILI). In addition—from a more fundamental standpoint an hPSC-hepatocyte differentiation model will likely aid in our fundamental understanding of human liver development.

Although hPSCs can differentiate towards the hepatocyte lineage and exhibit several liver-specific characteristics (i.e. expression of hepatocyte marker genes, albumin (ALB) secretion, glycogen storage, urea production; susceptibility to human specific hepatotropic infections, such as hepatitis virus B, C and E) [2–8], it is not yet possible to create fully mature PHHs from hPSCs. Indeed, PSC-derived hepatocyte progeny are termed fetal hepatocytes (FH) or hepatocyte-like cells (HLCs), as the cells continue to express for instance the fetal marker alpha-fetoprotein (AFP); remain glycolytic, and do not express mature type I & II detoxification enzymes [9–14]. Thus, one of the major goals of many groups developing hepatocyte progeny from hPSCs is to improve the differentiation system to create efficiently and reproducibly fully mature hepatocytes with phenotypic and metabolic similarities with PHHs.

Generation of hepatocytes involves sequential cell fate choices as a result of spatio-temporal modulation of the chromatin of gene regulatory regions. The histone methyltransferase, Enhancer of Zest Homolog 2 (EZH2), is the catalytic subunit of the polycomb repressive complex 2 (PRC2). Together with other PRC2 subunits (i.e. Embryonic Ectoderm Development (EED) and SUZ12), EZH2 mediates epigenetic silencing of target genes via trimethylation of histone H3 lysine residue 27 (H3K27me3) at specific regulatory loci [15–17]. Many of these genes are related to cell cycle checkpoints and differentiation, suggesting a major role of EZH2 in promoting cell proliferation and self-renewal [18,19]. Indeed, deletion of EZH2 in hPSC leads to compromised self-renewal and differentiation defects [20]. PRC2 is not necessary for maintaining ESC self-renewal, as each of the PRC2 components can be deleted without compromising the expression levels of pluripotent markers, such as OCT4 and NANOG [21,22]. Moreover, ESC lacking SUZ12, EED or EZH2 show aberrant de-repression of lineage-specific genes and are unable to properly differentiate. This is also partially due to the lack of repression of pluripotent genes during differentiation [21,22]. It has also been described that in hepatic stem/progenitor cells EZH2 has the capacity to block the differentiation towards hepatocytes [23], however we have shown that inhibition of EZH2, at a later time point of hepatocyte differentiation, decreased H3K27me3 in regulatory regions, but did not affect hepatocyte gene expression, and is therefore dispensable for the later stages of maturation of hESCs to a mature hepatocyte phenotype *in vitro* [24]. This suggests that temporary overexpression of EZH2 during the initial steps of the PSC-hepatocyte differentiation protocol, but not at later stages should improve the generation of mature hepatocytes from PSCs.

Here, we demonstrate that doxycycline inducible overexpression of *EZH2* from the *AAVS1* locus resulted in improved definitive endoderm formation from hPSCs and subsequent fetal hepatocytes generation. Surprisingly, despite doxycycline mediated *EZH2* overexpression between endoderm and hepatoblast stage of the differentiation protocol, transcript and protein levels of EZH2 decreased progressively from endoderm onwards. This was associated with an increased expression of micro (mi)RNAs that are known/predicted to suppress *EZH2* expression. In conclusion, we demonstrate that EZH2 plays an important role in hepatocyte differentiation and that its expression is tightly post-transcriptionally regulated.

## Materials and methods

### Cell lines and hESC differentiation to the hepatocyte lineage

The human embryonic kidney (HEK293) cell line was cultured in Dulbecco’s Modified Eagles’s Medium (DMEM, Invitrogen, USA) medium that contained 10% fetal bovine serum (FBS) and 1X penicillin/streptomycin (Invitrogen, USA). The hESC H9 line (WAO9, WiCell) was cultured on inactivated mouse embryonic fibroblasts (iMEF) as described [25]. Experiments were performed with approval from the Medical Ethics Committee (UZ Leuven, Gasthuisberg). Hepatocyte differentiation was done as described earlier with minor modifications [5,7,24,26]. EZH2 was induced in the cultures by administration of 5μg/ml doxycycline (Sigma-Aldrich) from day 0 to day 4 and day 8 by changing media completely every 2 days. All growth factors were purchased from PeproTech.

### Plasmid construct and generation of the master cell lines and Recombinase-Mediated Cassette 3xchange (RMCE) line

All constructs were fully sequenced before use. *EZH2* transcript variant 1 (TV1) fused to a myc epitope was obtained by RT-PCR from hESCS and was cloned into the *pZ*:*F3*‐*P P TetOn-F* [26] vector which contains the Tetracycline Response Element (TRE) from pTRIPZ (Open Biosystems). The “all‐in‐one” vector contains the *TRE* driving the expression of *Myc-EZH2* in reverse orientation to the *CAGGS m2rtTA* cassette. The master cell line and the RMCE line were generated as described [26]. *EZH2* transcript variant 1 (TV1) was cloned into pLVX-IRES-Hygro (Clontech) vector and used as positive control.

### Immunofluorescence and flow cytometry

hESCS and/or differentiated cells were grown on glass slides and fixed with 4% paraformaldehyde (PFA), permeabilized with 0.2% Triton X-100 in PBS, blocked with 5% normal donkey serum (Jackson Laboratory), and stained overnight at 4°C with OCT4 (0.4 μg/mL, Santa Cruz Sc-8628), TRA-1-60 (1 μg/ml, Millipore-Chemicon MAB4360), SOX17 (5 μg/mL, R&D AF1924), HNF4A (5 μg/mL, Abcam Ab41898), ALB (2.5 μg/mL, Dako A0001) and AAT (3.95 μg/mL, Dako A0012), or the relevant isotype controls in Dako diluent (Dako). Secondary antibodies were used at 1:500 dilution (species-specific AF555-conjugated immunoglobulin G, Alexa Fluor, Molecular Probes) and nuclei were visualized using Hoechst (Sigma-Aldrich). Signals were detected with an Axioimager.Z1 microscope and analyzed with the Axiovision software (Zeiss). The percentage of SOX17 and HNF4α positive cells was manually counted on five representative 10× images. For all pictures, the percentage of positive cells was contoured above the isotype level and three different differentiations were averaged. The histone modification H3K27me3 was stained and quantified as described [24]. For CXCR4/cKIT flow cytometry, cells were detached at day 4 with trypsin 0.05% and stained with 1μg/mL anti-CXCR4-PE and 2μg/ml anti-cKIT-APC antibody for 15 minutes at room temperature. Afterwards cells were washed and analyzed by flow cytometry analysis using a FACS-Canto (BD).

For intracellular AAT flow cytometry staining, a single cell suspension was made by liberase treatment (Roche) followed by fixation with 4% PFA. Next, cells were permeabilized with 0.1% saponin and blocked with 10% goat serum (Dako). Afterwards cells were stained with 0.0625μg/200μL/10^6 cells anti-AAT antibody (Dako) or a rabbit IgG isotype control (BD Pharmingen) for 1h at RT. A secondary Alexa Fluor 647 antibody (1:1500) (Invitrogen) was used for 30 minutes at RT. Cells were analyzed by flow cytometry analysis using a FACS-Canto (BD) and analyzed with FACS Diva Software (BD Biosciences).

### RNA extraction and quantitative reverse-transcription PCR (qRT-PCR)

For gene expression analysis, RNA was isolated from differentiated progeny cells by the GenElute Mammalian Total RNA Miniprep Kit (Sigma-Aldrich) following manufacturer’s procedures. Genomic DNA was eliminated using the On-Column DNase I Digestion kit (Sigma-Aldrich). The Superscript III First-Strand synthesis system (Invitrogen) was used for subsequent cDNA synthesis. qPCR was performed with the Platinum SYBR green qPCR supermix-UDG kit (Invitrogen) in a ViiA 7 Real-Time PCR instrument (Thermo Fisher Scientific, Waltham, MA). Glyceraldehyde-3-phosphate dehydrogenase (GAPDH) was used as the housekeeping gene for normalization. Sequences of qRT-PCR primers are listed in Table 1. Relative expression to GAPDH was calculated as 2‐ΔCt and relative gene expression as fold change was calculated as 2‐ΔΔCt.

**Table 1:** Gene expression primers.

Gene	Forward	Reverse
*GAPDH*	TCAAGAAGGTGGTGAAGCAGG	ACCAGGAAATGAGCTTGACAAA
*EZH2*	TGGGAAAGTACACGGGGATA	CAGGATCGTCTCCATCATCA
*EZH2 total*	TGATGCCCTGAAGTATGTCG	GGGTTCCTTCCGGTATTGTC
*EZH1*	TTTTCATGCCACCCCTAATG	AGCATGGCATACTCCTTTGC
*SUZ12*	AAACGAAATCGTGAGGATGG	CCATTTCCTGCATGGCTACT
*EED*	GGCAATATTTGGAGGCGTAG	GAATGATCCATACCACAGGACA
*RING1B*	GTGCAGACAAACGGAACTCA	TCGAGGTGAAACCACAATTTC
*BMI1*	TGTTCGTTACCTGGAGACCA	TTTTGAAAAGCCCTGGAACT
*OCT4*	TCGAGAAGGATGTGGTCCGA	GCCTCAAAATCCTCTCGTTG
*NANOG*	CCTGTGATTTGTGGGCCTG	GACAGTCTCCGTGTGAGGCAT
*FOXA2*	AGGAGGAAAACGGGAAAGAA	GGTGCTTGAAGAAGCAGGAG
*EOMES*	AACAACACCCAGATGATAGTC	TCATAGTTGTCTCTGAAGCCT
*MIXL1*	GGATCCAGGTATGGTTCCAG	CATGAGTCCAGCTTTGAACC
*GOOSECOID*	TCTCAACCAGCTGCACTGTC	CCAGACCTCCACTTTCTCCTC
*BRACHYURY*	ACCCAGTTCATAGCGGTGAC	AAGCTTTTGCAAATGGATTG
*SOX17*	CGCTTTCATGGTGTGGGCTAAGGACG	TAGTTGGGGTGGTCCTGCATGTGCTG
*CXCR4*	CACCGCATCTGGAGAACCA	GCCCATTTCCTCGGTGTAGTT
*HNF4A*	ACTACGGTGCCTCGAGCTGT	GGCACTGGTTCCTCTTGTCT
*HNF1A*	ACACCTCAACAAGGGCACTC	TGGTAGCTCATCACCTGTGG
*HNF3B*	AGGAGGAAAACGGGAAAGAA	GGTGCTTGAAGAAGCAGGAG
*HNF3G*	ATTCTCTCTGGCATGGGTTG	AAATTCCCCACACCCTAACC
*HNF6*	AAATCACCATTTCCCAGCAG	ACTCCTCCTTCTTGCGTTCA
*GSTA1*	TCTGCCCGTATGTCCACCT	GCTCCTCGACGTAGTAGAGAAGT
*APOA1*	TGGATGTGCTCAAAGACAGC	TCACCTCCTCCAGATCCTTG
*SRBI*	TGAACTGCTCTGTGAAACTG	AATAGCATTTCTCTTGGCTCC
*GSTp*	CCCTACACCGTGGTCTATTTCC	GAGGCTTTGAGTGAGCCCT
*AFP*	TGAGCACTGTTGCAGAGGAG	GTGGTCAGTTTGCAGCATTC
*PROX1*	TCACCTTATTCGGGAAGTGC	GGAGCTGGGATAACGGGTA
*ALBUMIN*	ATGCTGAGGCAAAGGATGTC	AGCAGCAGCACGACAGAGTA
*AAT*	AGGGCCTGAAGCTAGTGGAT	TCCTCGGTGTCCTTGACTTC
*MRP2*	CGATATACCAATCCAAGCCTC	GAATTGTCACCCTGTAAGAGTG
*NTCP*	ATCGTCCTCAAATCCAAACG	CCACATTGATGGCAGAGAGA
*PEPCK*	AAGAAGTGCTTTGCTCTCAG	CCTTAAATGACCTTGTGCGT
*CKIT*	TCAGTGCATAACAGCCTAATCTC	GTTCTGCTCCTACTGCTTCG
*PXR*	TTCCGGGTGATCTCGCA	ACTGGCTATCACTTCAATGTCA
*CAR*	GCTCCTGCTGTGCTTCGT	GCATGGCAGATAGGCAGTTT
*P300*	CCAGATGGGAGGACAAACAG	CCAATCTGCTGTCCAGGATT
*CEBPA*	AAAGGGGTGGAAACATAGGG	GGAGAGGCGTGGAACTAGAG
*CEBPB*	CAACAAGCCCGTAGGAACAT	ATTCTCTCTGGCATGGGTTG
*PDX1*	TCCACCTTGGGACCTGTTTA	GTGTGTTAGGGAGCCTTCCA
*NEUROD1*	CTCGGACTTTTCTGCCTGAG	GTGGAAGACATGGGAGCTGT
*NGN3*	TCTCTATTCTTTTGCGCCGG	CTTGGACAGTGGGCGCAC
*NKX6*.*1*	CCATCTTCTGGCCCGGAGTGA	CTTCCCGTCTTTGTCCAACAA
*PAX6*	CCCAAGAGCAAATTGAGGCCC	CTCTTCTCCATTTGGCCCTTCGA
*INK4A*	GAAGGTCCCTCAGACATCCCC	CCCTGTAGGACCTTCGGTGAC
*INK4B*	ACTAGTGGAGAAGGTGCGAC	GCCCATCATCATGACCTGGA
*ARF*	CCCTCGTGCTGATGCTACTG	ACCTGGTCTTCTAGGAAGCGG
*P21*	GGCAGACCAGCATGACAGATTTC	CGGATTAGGGCTTCCTCTTGG
*CYP1A2*	CAGCTCTGGGTCATGGTTG	CCTCCTTCTTGCCCTTCAC
*CYP2C9*	GTTTCTGCCAATCACACGTTC	CTGCAGTTGACTTGTTTGGAG
*CYP2A6*	GTTGTACATCTGCTCTGTGTTC	GTGGCCTTGCTGGTCTG
*CYP3A7*	CTCCCTGAAAGGTTCAGTAAA	AAGAAGTCCTCCAAAGCG

### microRNA (miRNA) analysis

Total RNA samples, which include RNA from approximately 18 nucleotides (nt), were extracted and purified using miRNeasy Mini Kit (QIAGEN, ID: 217004) accordingly to the manufacturer’s procedures.

Then, 1μg of RNA was polyadenylated and reverse transcribed (RT) using NCodeTM miRNA First-Strand cDNA Synthesis and qRT-PCR Kit (Invitrogen, MIRQ-10) with a universal primer. For the quantification miRNAs, real-time PCR for each microRNA assay was carried out in 10μl reaction mixture included 2 μl of diluted RT product, 2× SYBR green, and 0.5 μM forward primer and reverse primer. The reaction was incubated in ViiA 7 Real-Time PCR instrument (Thermo Fisher Scientific, Waltham, MA) in 384-well plates at 95°C for 10 min, followed by 45 cycles of 95°C for 15 sec and 60°C for 30 sec. U6 was used as the housekeeping gene for normalization. Sequences of miRNA primers are listed in Table 2. Relative expression to U6 was calculated as 2‐ΔCt and relative gene expression as fold change was calculated as 2‐ΔΔCt.

**Table 2:** miRNAs expression primers.

Mature name	Primer
hsa-miR-101-3p	CCGGTACAGTACTGTGATAACTGAA
hsa-miR-138-5p	AGCTGGTGTTGTGAATCAGGC
hsa-miR-214-3p	TGCCTGTCTACACTTGCTG
hsa -miR-124-5p	CGTGTTCACAGCGGACCTTG
hsa -miR-31-5p	AGGCAAGATGCTGGCATAGC
hsa -miR-98-5p	GAGGTAGTAAGTTGTATTGT
hsa-miRNA-125b-5p	TCCCTGAGACCCTAACTTG
has-miR-139-5p	TACAGTGCACGTGTCTCCAGT
hsa-miRNA-181a-5p	AACATTCAACGCTGTCGGTG
hsa-miR-181b-5p	AACATTCATTGCTGTCGGTGG
has-miRNA-200b-3p	TAATACTGCCTGGTAATGAT
has-miR-217	TACTGCATCAGGAACTGATTGG
U6	CTCGCTTCGGCAGCACA

### Protein extracts, immunoblotting, and antibodies

For protein expression analysis, cells from differentiated progeny cells were lysed in 2x Laemmli Buffer (100 mM Tris-Cl (pH 6.8), 2% SDS, 20% glycerol and 4% β-mercaptoethanol added fresh) in PBS at 95°C for 10 min. Protein extracts were separated using a SDS-PAGE gel and transferred onto a nitrocellulose membrane (Whatman International Ltd, Maidstone, UK). Immunoblots were incubated with primary antibodies against EZH2 (Purified Mouse, BD dil. 1:100), RING1B (dilution 1:1000, Santa Cruz Biotech sc-101109), GATA4 (dilution 1:400, Santa Cruz Biotech sc-25310) and GAPDH (dilution 1:2000, Santa Cruz Biotech sc-47724) as loading control. Subsequently, HRP conjugated secondary antibodies against rabbit and mouse IgG were procured from Jackson Immunologicals (West Grove, PA). Immuno-reactive bands were then visualized using Super Signal® West Pico chemi-luminescent substrate (Thermo Scientific, Rockford, IL).

### Chromatin immunoprecipitation (ChIP)

ChIP was performed using 1x10^6 cells per H3K27me3 histone modifications. IPs were processed as described before [27]. Dynabeads (Life Technologies) were added and DNA purified using QIAquick PCR purification kit (Qiagen). Purified DNA was analyzed by qPCR. ChIP-grade ABs used, ChIP-qPCR data analysis and primers sequences for GAPDH, MYOD1, HOXD11, ALBUMIN and AAT are described in [24]. SOX17 5’-AGGTCACCCACCACTGAAAC-3’ and 5’-GAACATACCGAGCGTCCATT-3’, FOXA2 (5’-AATCTGGGCTCACAGGCTAA-3’ and 5’-TGTCTTCCAGAGGGACTGCT-3’) and CXCR4 5’- TCCAGACCTGGGAATGCTAC-3’ and 5’- GTTGGAAGCTTGGCCCTACT-3’.

### Albumin ELISA

Enzyme-linked immunosorbent assay (ELISA) for ALBUMIN was performed according to the manufacturer’s procedure (Bethyl, Montgomery, TX). Briefly, at day 16 of the hepatocyte differentiation protocol, supernatant was collected and incubated as described in [5].

### DNA methylation by bisulfite sequencing

Genomic DNA of the cells was extracted using the QIAamp DNA Mini kit (QIAGEN) and 500ng DNA were used to perform the bisulfite conversion with EpiTect Bisulfite Kit (QIAGEN), according to the manufacturer’s instructions and the following cycling process: [95°C, 30 sec– 50°C, 1 hour] X16–10°C as previously described [28]. Regular PCR was used to amplify specific fragments using 10‐20 ng of converted DNA using forward 5’-TTGTATTTTAGTTTGGATAATTAGAG-3’ and reverse 5’-AAAAACCAAATTTAAACCAATTCAA-3’ primers. The PCR products were cloned into a p‐GEM easy vector (Promega) and 15‐20 clones were sequenced. The methylation rate of the CpG pairs was quantified using QUMA software.

## Results

### The expression of EZH2 decreases during hepatocyte differentiation

**Fig 1:**
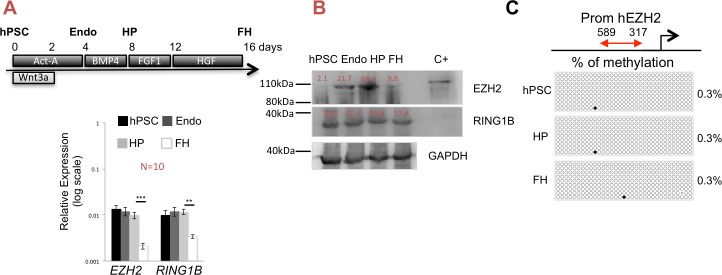
EZH2 expression during hepatocyte differentiation. **A.** Upper part, differentiation protocol of hPSCs into hepatocytes-like cells. hESC were harvested and plated at ±7 x 10^4^ cell/cm^2^ and differentiated with a combination of growth factors: 50ng/ml of Activin-A and Wnt3a for the first 2 days and 50ng/ml Activin-A until day 4, followed by BMP4 50 ng/ml from day 4 until day 8, FGF1 50 ng/ml from day 8 until day 12, and HGF 20 ng/ml from day 12 until day 16. Differentiated progeny of the hESC line were harvested on day 4 (endo_d4, definitive endoderm cells), on day 8 (HP_d8, hepatoblast progenitor) and on day 16 (FH_d16, fetal hepatocyte). Bottom part, transcript expression levels of *EZH2* and *RING1B* in hPSCs, endo, HP and FH stages. *GAPDH* was used as a control. Data as mean ± SEM of n ≥ 3 IEs. *** p < 0.001 by Student’s t test. **B.** Western blot analysis of EZH2, RING1B and GAPDH on hPSCs, endo, HP and FH stages. GAPDH was used as loading control. HEK293T transiently transfected with pLVX-IRES-Hygro-hEZH2 cells were used as positive control. Signals were quantified and indicated as % to loading control. **C.** Schematic representation of the *EZH2* regulatory region. Approximately 270bp (arrow in front of the TTS) in a CpG island were bisulfite sequenced in undifferentiated hPSC, HP and FH. Percentages of the results of bisulfite sequencing of the regions are at right side of the figure for each stage.

We first evaluated the transcript levels of the polycomb group (PcG) genes *EZH2*, *EZH1*, *SUZ12*, *EED*, *RING1B* and *BMI1* in hPSCs, definitive endoderm (endo_d4), hepatic progenitors (HP_d8) and fetal hepatocytes (FH_d16) derived from hPSCs (Fig 1A, upper part) [5,7,24,26]. An intermediate point, hPSC-derived hepatoblast at day 12, has not been analyzed in this manuscript. *EZH1*, *SUZ12* and *EED* were expressed at constant levels throughout the hepatocyte differentiation protocol, whereas transcript levels for *RING1B* decreased and *BMI* increased by day 16 (S1 Fig). Expression of *EZH2* was gradually decreasing during the progression of the hepatocyte differentiation protocol (day 4, endo and day 8, HP), and significantly decreased by day 16, FH (Fig 1A, bottom part and S1 Fig). Different from the RT-qPCR results (Fig 1A, bottom part and S1 Fig), no changes in RING1B protein levels were observed by western blot throughout differentiation (Fig 1B). However, western blot analysis for EZH2 protein during hepatocyte differentiation demonstrated an increase in EZH2 between day 0 and day 4 of differentiation, and a decrease of EZH2 from day 8 to day 16 of differentiation (Fig 1B). The low levels of EZH2 protein in undifferentiated hPSCs were not in accordance with the transcript levels detected by RT-qPCR (Fig 1A, bottom part). Thus, during HLCs generation from PSCs, there appears to be a dynamic regulation of EZH2 protein.

To investigate whether epigenetic modifications modulate *EZH2* gene expression during hPSC-HLCs differentiation, we studied the role of DNA methylation of the regulatory region upstream of the transcription start site (TSS) of EZH2, containing 33 CpG sites in a CpG island (Fig 1C, upper part), in hPSCs, HP (day 8) and FH (day 16). Bisulfite sequencing demonstrated DNA hypomethylation of this region at all 3 steps of differentiation analyzed (Fig 1C, bottom part). These results indicate that expression levels of EZH2 are not regulated by DNA methylation in the region of the TSS of *EZH2*, but suggest that *EZH2* mRNA could be regulated by other epigenetic modifications, as the protein levels are most likely regulated by posttranslational modifications.

### Definitive endoderm (DE) formation is improved by EZH2 overexpression

**Fig 2:**
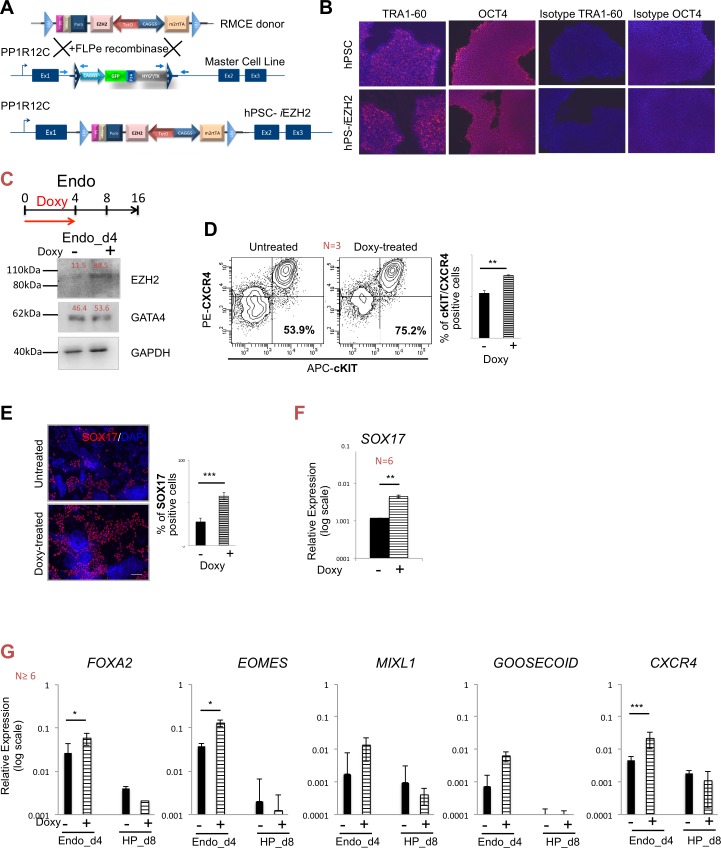
hESC-inducible EZH2 (hPSC- *i*EZH2) cell line and definitive endoderm formation. **A.** The original master cell line flanked by heterotypic FRT sequences and resulting RMCE line (Recombinase-Mediated Cassette Exchange, hPSC-*i*EZH2) are depicted. RMCE donor vector *pZ*:*F3-P TetOn hEZH2* for inducible expression was introduced by flippase (additional details in the Supplemental Information). **B.** Expression of pluripotency markers (TRA-1-60 and OCT4) of a representative hPSC-*i*EZH2 clone and wild-type hPSCs by immunocytochemistry. Right: isotype control of TRA-1-60 and OCT4. **C.** Upper part, directed differentiation of hPCS-*i*EZH2 toward endoderm (endo_d4) with addition of 5μg/ml doxycycline (doxy) in the first 4 days of differentiation (red arrow). Bottom part, protein analysis of untreated and doxy treated EZH2 induced cells (doxy -/+) using EZH2, GATA4 and GAPDH antibodies. GAPDH was used as loading control. Signals were quantified and indicated as % to loading control. **D.** Double extracellular staining for CXCR4 (PE channel) and cKIT (APC channel) measured by FACS in untreated and doxy treated EZH2 induced cells (doxy-treated) at definitive endoderm stage on day 4. Right: the percentage of cells co-expressing CXCR4 and cKIT. Data as mean ± SEM of n ≥ 3 IEs. ** p < 0.01 by Student’s t test. **E.** Representative immunofluorescence images for SOX17 (red signal) in untreated and EZH2 induced cells (doxy-treated) at day 4 of differentiation. EZH2 induced cells showed significantly high levels of SOX17 expression. Around 58% of positive cells were counted in EZH2 doxy induced cells, right part. Nuclei are staining with DAPI (blue). Data as mean ± SEM of n ≥ 3 IEs. *** p < 0.001 by Student’s t test. **F.** mRNA expression profile of the endoderm marker *SOX17* in untreated (-) and EZH2 doxy induced cells (+) at endo_d4. **G.** mRNA expression profile of untreated (-) and EZH2 doxy induced cells (+) at endo_d4 and HP_d8 of endoderm markers *FOXA2*, *EOMES*, *MIXL1*, *GOOSECOID* and *CXCR4*. Relative gene expression to GAPDH. Data as mean ± SEM of n ≥ 3 IEs.

We recently described an efficient and very fast method to introduce doxycycline (doxy) inducible transgenes in hPSCs, by recombinase mediated cassette exchange (RMCE) in a flippase recognition target (FRT) flanked cassette in the adeno-associated virus integration site-1 (*AAVS1)* locus of hPSCs [26]. This allowed us to study the effect of inducible *EZH2* overexpression on the differentiation potential of hPSCs towards HLCs. We inserted the amplified, human *EZH2* transcript variant 1 (h*EZH2* tv1, NM_004456) sequence into the “all-in-one” inducible RMCE donor vector that contains the tetracycline response element *(TRE)* driving the expression of the transgene in reverse orientation to the *CAGGS m2rtTA* cassette (Fig 2A) and flanked by FRT sites. The cassette was introduced by flippase-mediated cassette exchange in the *AAVS1* as described earlier [26]. The ihEZH2 inducible cell line (hPS-*i*EZH2) maintained the typical pluripotency characteristics of hPSCs as the expression of pluripotency genes OCT4 and TRA1-60 was comparable to wild-type hPSCs (Fig 2B).

**Fig 3:**
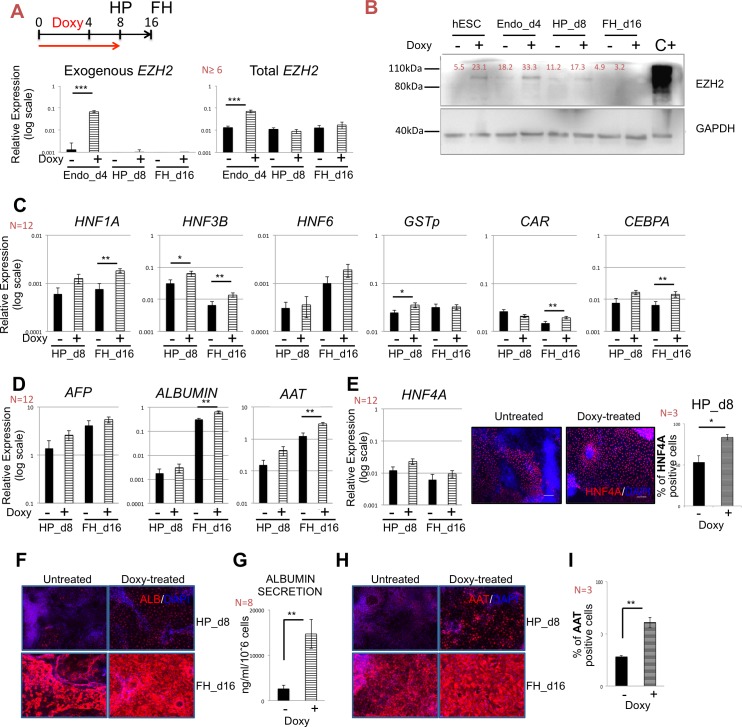
EZH2 regulation and fetal hepatocytes differentiation. **A.** Upper part, directed differentiation of hPCS-*i*EZH2 toward fetal hepatocytes (FH_d16) with addition of 5μg/ml doxycycline (doxy) in the first 8 days of differentiation (red arrow). Bottom part, relative gene expression of the *EZH2* transgene (Exogenous *EZH2*) and both endogenous and exogenous EZH2 (Total *EZH2*) at endo_d4, HP_d8 and FH_d16 in untreated (-) and EZH2 doxy induced cells (+). Relative gene expression to GAPDH and same scale bar. Data as mean ± SEM of n ≥ 3 IEs. **B.** Western blot for EZH2 and GAPDH in untreated (-) and EZH2 doxy induced cells (+) at hPSC, endo_d4, HP_d8 and FH_d16. Positive control: HEK293T cells transiently transfected with pLVX-IRES-Hygro-hEZH2. The molecular weight size marker was cropped from the gel. Signals were quantified and indicated as % to loading control. **C.** Relative expression (to *GAPDH*) of hepatic markers *HNF1A*, *HNF3B*, *HNF6*, *GSTp* and transcription factors *CAR* and *CEBPA* in HP_d8 and FH_d16 untreated (-) and EZH2 doxy induced cells (+). Data as mean ± SEM of n ≥ 3 IEs. * p < 0.05 and ** p < 0.01. **D.** Relative gene expression (to *GAPDH*) of hepatic marker genes *AFP*, *ALBUMIN and AAT* in HP_d8 and FH_d16 untreated (-) and EZH2 doxy induced cells (+). Data as mean ± SEM of n ≥ 3 IEs. * p < 0.05, ** p < 0.01. **E.** Left part, relative gene expression (to *GAPDH*) of hepatic transcription factor HNF4A in HP_d8 and FH_d16 untreated (-) and EZH2 doxy induced cells (+). Right part, representative immunofluorescence images for HNF4A (red signal) on day 8 for cells treated without (untreated) or with doxy (doxy-treated). Nuclei are staining with DAPI (blue). On the right, more than 80% of HNF4A positive cells on HP_d8 EZH2 doxy induced cells were counted. Data as mean ± SEM of n ≥ 3 IEs. * p < 0.05. **F.** Immunofluorescence staining for ALBUMIN (red signal) at HP (day8) and FH (d16) in untreated (-) and EZH2 induced cells (+) (left part). Nuclei are staining with DAPI (blue). Data as representative images of n = 2 IES. **G.** ELISA for ALBUMIN secretion on FH_d16 of the hepatocyte differentiation protocol. EZH2 doxy induced cells (+) secrete significant amounts of albumin compared to untreated (-) cells. Data as mean ± SEM of n ≥ 3 IEs. ** p < 0.01. **H.** Immunofluorescence staining for AAT (red signal) at HP (day 8) and FH (day 16) showed abundant expression of the hepatocyte protein in doxy-treated cells compared to the untreated (left part). Nuclei are stained with DAPI (blue). Data as representative images of n = 2 IES. **I.** Intracellular flow cytometry analysis for AAT demonstrated that more then around 60% of EZH2 doxy induced cells (+) progeny were positive for AAT. Results represent the mean of three independent experiments ± SEM. ** p < 0.01.

To further investigate the regulatory role of EZH2 during hPSC differentiation into definitive endoderm (endo_d4) we induced the expression of EZH2 with doxy from day 0 to day 4 in hepatocytes differentiation protocol (Fig 1A, upper part). Both western blot (Fig 2C) and RT-qPCR (Figs 3A, bottom part and 5A) analysis confirmed that addition of 5μg/ml doxy to the differentiation medium from day 0 to day 4 significantly induced the expression of *EZH2* compared to the untreated control. In response to the higher levels of *EZH2*, a significantly more homogenous population of definitive endoderm cells was generated on day 4, demonstrated by flow cytometry analysis for CXCR4/cKIT double positive cells (75.2±0.91% in the presence of doxy and 53.9±3.29% in the absence of doxy, n = 3, p = 0.0172) (Fig 2D). This coincided with a significant increase in SOX17 positive cells (57.94±4.31% with doxy vs. 27.64±4.32% without doxy, n = 2, p = 0.00009), demonstrated by immunostaining (Fig 2E) and also by RT-qPCR (Fig 2F). In addition, transcripts for the definitive endoderm markers, *FOXA2*, *EOMES*, *MIXL1*, *GOOSECOID* and *CXCR4*, were up regulated in doxy-treated (Fig 2G) compared to untreated control cells. As expected, 8 days post differentiation (HP_d8), expression of the definitive endoderm markers *FOXA2*, *EOMES*, *MIXL1*, *GOOSECOID* and *CXCR4* decreased, and no differences were seen between doxy-treated or untreated cells (Fig 2G). RT-qPCR also demonstrated that the key pluripotency markers *OCT4* and *NANOG* decreased over-time (S2 Fig). In conclusion, EZH2 induction significantly improved definitive endoderm formation.

### Regulation of EZH2 overexpression during hepatocyte differentiation

To address if induction of EZH2 also improved the generation of day 16 fetal hepatocytes from hPSCs, we differentiated hPS-*i*EZH2 with or without 5μg/ml doxycycline (doxy) from the start of differentiation until day 8 (Fig 3A, upper part). EZH2 was significantly induced in hPSC-progeny on day 4. Despite continued addition of doxy until day 8, *EZH2* mRNA levels were almost zero beyond day 4, as analyzed by RT-qPCR with primers selective for the *EZH2* variant 1 transgene transcript (Exogenous *EZH2*, Fig 3A, left) and primers that recognized both endogenous and transgenic *EZH2* mRNA (*Total EZH2*, Fig 3A right). Expression of *EZH1* mRNA did not change throughout differentiation and was not affected by doxy treatment (S2 Fig). Western blot confirmed that EZH2 protein levels were higher in doxy treated compared with control cells on day 4, but were no longer higher on day 8, and were not detectable on day 16 (Fig 3B). Thus, despite addition of doxy between day 0 and day 8, *EZH2* transcript and protein levels decreased progressively from day 4 onwards, suggesting that post-transcriptional regulation of EZH2 during differentiation might be responsible for *EZH2* mRNA down-regulation.

We also examined the effect of EZH2 overexpression on the differentiation of hPSCs towards fetal hepatocytes on day 16. Although *EZH2* transcripts and protein were only enhanced during the initial 4 days of differentiation, we observed a significant increased expression of *HNF3B* and *GSTp* on day 8, and a significant increase in *HNF1A*, *HNF3B*, *CEBPA*, and *CAR* transcripts on day 16 (Fig 3C). We also found significantly increased transcript levels for *ALB* and *AAT*, two genes expressed in mature hepatocytes (Fig 3D). *AFP*, a typical hepatoblast/fetal hepatocyte gene, is continuously expressed as expected for the fetal nature of the hPSC-derived hepatocytes among samples (Fig 3D). We also monitored the expression of some additional fetal markers (*CYP3A7*, *CYP1A2* and *CKIT*) and mature hepatocyte genes on day 8 and day 16 (*CYP2A6*, *CYP2C9*, *CEBPB*, *GSTA1*, *APOA1*, *NTCP*, *SRBI*, *P300*, *MRP2*, *PROX1*, *PEPCK*, *PXR* and *HNF3G*). Only the levels of *CYP3A7*, *CYP2A6* and *CYP2C9* were significantly influenced by overexpression of EZH2 between day 0 and day 4 (S3 Fig).

On HP_d8, levels of *HNF4A* mRNA were not affected by addition of doxy (Fig 3E, left), but immunostaining demonstrated presence of significantly more HNF4A positive cells in the doxy treated cells (83.5±3.37% with doxy vs. 53.3±7.58% without doxy, n = 3, p = 0.0409) (Fig 3E, right). On HP_d8 and FH_d16, immunostaining (Fig 3F and 3H) suggested that more doxy-treated progeny stained positive for ALBUMIN and AAT (n = 2). Consistently, ALBUMIN secretion by doxy treated progeny was significantly increased on day 16 (Fig 3G), and flow cytometry analysis demonstrated that significantly more doxy treated cells stained positive for AAT (60.9 ±8.51% with doxy vs. 27.7 ±2.31 without doxy, n = 4, p = 0.015).

As EZH2 also plays a role in pancreas versus liver fate choice from endoderm [29,30], we also assessed the expression of *PDX1*, *NGN3*, *NEUROD1*, *NKX6*.*1* and *PAX6* during differentiation. We did not observe increased levels of these transcripts (S3 Fig) on day 8 and day 16 hPSC progeny in the presence of doxy compared with cultures without doxy, indicating that the improved hepatocyte specification as a result of EZH2 induction was specific.

Thus, enhanced expression of EZH2 between day 0 and day 4 significantly increased ALBUMIN secretion and significantly increased AAT expressing cells on d16 of differentiation, while not affecting pancreatic fate commitment. Interestingly, improved hepatic commitment on day 16 occurred despite the fact that EZH2 transcript and protein levels were only significantly elevated between day 0 and day 4.

**Fig 4:**
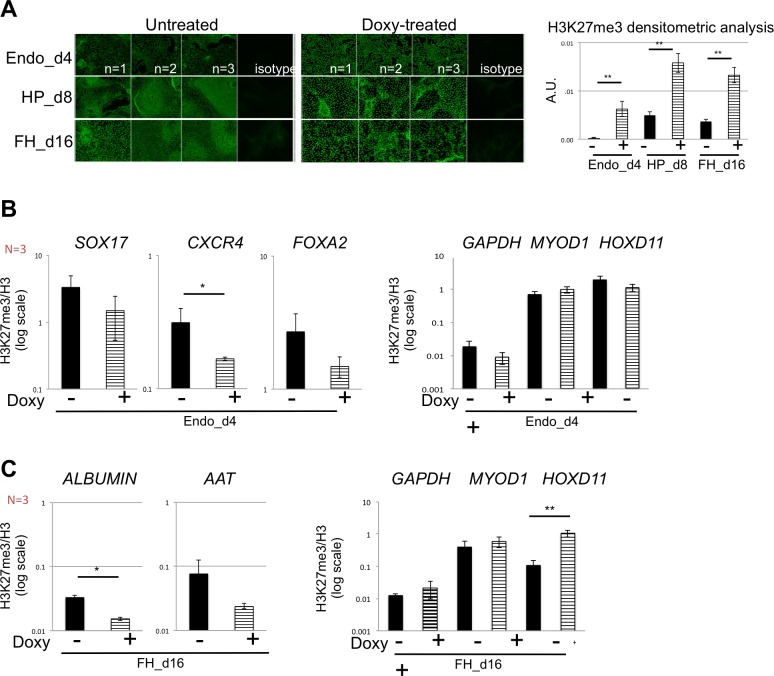
Total H3 levels and H3K27me3 levels on endodermal and hepatocyte genes. **A.** Left, representative immunofluorescence image for H3K27me3 (green signal) of three different differentiations (n = 1, 2 and 3) at endo_d4, HP_d8 and FH_d16 stages in untreated and doxy-treated cells. Right, the fluorescence of H3K27me3 signal was digitally quantified (additional details in the Supplemental Information). Results represent the mean of three independent experiments ± SEM and displayed as arbitrary units (A.U.). ** p < 0.01. **B.** Histone modification levels of H3K27me3 at SOX17, CXCR4 and FOXA2 (left part) and GAPDH, MYOD1 and HOXD11 promoter (right part) at endo_d4 in untreated and doxy-treated cells. Results represent the mean of three independent experiments ± SEM. * p < 0.05. **C.** Histone modification levels of H3K27me3 at ALBUMIN and AAT (left part) and GAPDH, MYOD1 and HOXD11 promoter (right part) at FH_d16 in untreated and doxy-treated cells. Results represent the mean of three independent experiments ± SEM. * p < 0.05, ** p < 0.01.

Upon doxy-mediated induction of *EZH2*, H3K27me3 was significantly increased on day 4, day 8 and d16 hPSC progeny cells as was shown in Fig 4A (by immunofluorescence, left, and by densitometric analysis, right), while H3K27me3 significantly decreased on the endodermal markers (Fig 4B, left). The level of H3K27me3 on GAPDH, MYOD1 and HOXD11 remained unchanged between treated and doxy-treated cells on day 4 (Fig 4B, right). Moreover, a significant reduction of H3K27me3 on ALBUMIN (Fig 4C, left) and an increase on HOXD11 on day 16 (Fig 4C, right), a typical EZH2 target gene, suggested that EZH2 induction probably leads to the repression of not-hepatocyte genes while inducing endodermal and hepatocyte genes.

### Are miRNAs responsible for *EZH2* mRNA degradation?

**Fig 5:**
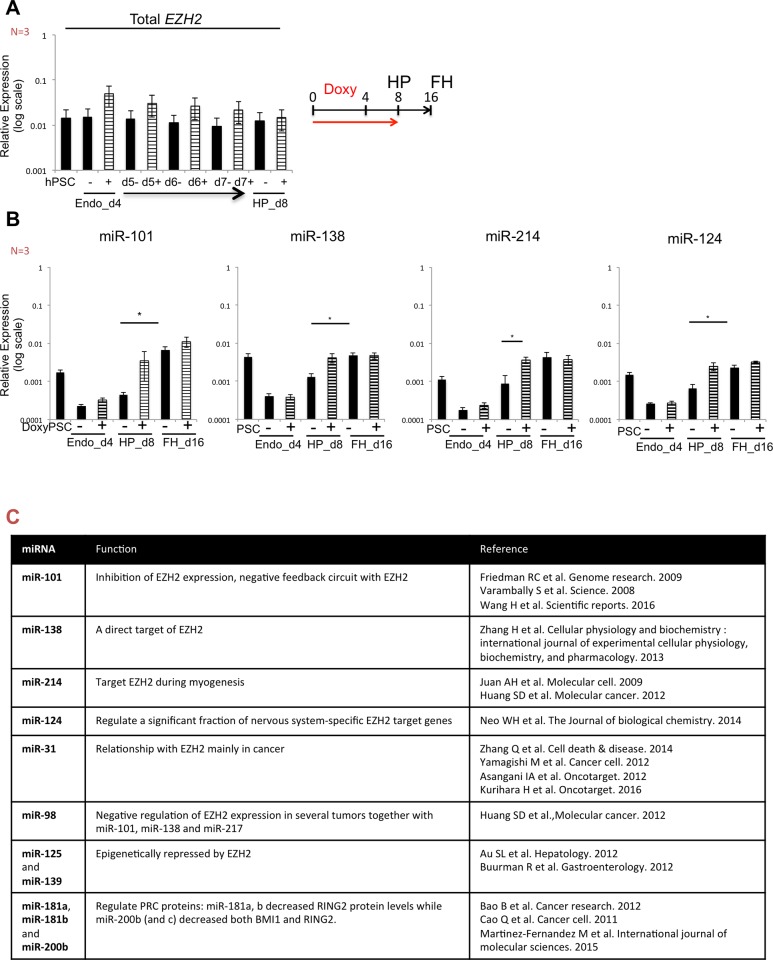
miRNA and EZH2 mRNA degradation. **A.** Relative gene expression (to *GAPDH*) of endogenous and exogenous *EZH2* (Total *EZH2*) daily during the transition from endoderm stage (day 4, endo_d4) to hepatoblast progenitor (day 8, HP_d8) in untreated (-) and EZH2 doxy induced cells (+). Data as mean ± SEM of n = 3 IEs. **B.** Relative expression of miR-101, miR-138, miR-214 and miR-124 during hepatocytes differentiation from hPSC-*i*EZH2 cell line doxy induced the first 8 days of differentiation. Relative gene expression to U6. Data as mean ± SEM of n = 3 IEs. **C.** Name, functions and reference papers of the miRNAs analyzed.

We have previously shown that inducible transgenes remained stably expressed throughout the differentiation when induction started from day 0 [26]. To further understand the kinetics of the decreased *EZH2* expression from day 4 onwards despite constant administration of doxy until day 8 (Fig 5A, right), we monitored *EZH2* transcript levels daily between day 4 and day 8. We demonstrated that *EZH2* mRNA levels in doxy-treated cells gradually decreased between day 4 and day 8 (Fig 5A, left) while levels in untreated cells remained constant, suggesting some kind of post-transcriptional regulation mechanism that limited the total amount of EZH2. We therefore examined the levels of miRNAs known/predicted to have a link to EZH2 (see Fig 5C). miR-101 has been identified to directly target *EZH2*, acting as an *EZH2* silencer involved in a negative feedback circuit with *EZH2* [31,32]. In addition, miR-138 was recently reported to directly target *EZH2* [33] and miR-214 has been shown to directly target *EZH2* during myogenesis [34]. To identify additional candidate miRNAs that might target *EZH2*, we used data from published studies [35,36] and examined the online database miRDB (http://mirdb.org/miRDB/) for miRNA target prediction (S4 Fig). We monitored expression of 12 know/predicted miRNAs targeting EZH2. As shown in Fig 5B and S4B Fig, expression levels of all miRNAs analyzed were lower in day 4 hPSCs progeny (±doxy) compared with undifferentiated hPSCs. Transcript levels of miR-101, miR-138, miR-214 and miR-124 were significantly higher in day 16 progeny compared to day 8 progeny, which was more pronounced (albeit not significant) for cultures supplemented with doxy (Fig 5B). The other candidate *EZH2* binding miRNAs displayed a very similar pattern of expression: for example, the expression kinetics of miR-139 was similar to that of miR-101, and the expression pattern of miR-31 and miR-200b was comparable to that of miR-138 throughout differentiation (S4 Fig). miR-98, miR-125 and 181a were detectable only on day 16, and levels were similar between doxy treated and untreated cells. miR-217 was the only EZH2 binding miRNA that could not be detected at any point during differentiation (S4 Fig).

These studies demonstrated that miRNAs known/predicted to interact with *EZH2* are highly up regulated during HLC differentiation and might explain a possible mechanisms for the loss of *EZH2* mRNA and EZH2 protein expression from day 4 onwards, despite the transgenic overexpression of *EZH2* until day 8 of differentiation.

## Discussion

hPSCs can differentiate into all somatic cell types of the human body, including hepatocytes. Thus, hPSC-derived hepatocytes are an attractive alternative to PHHs to test the safety, efficacy, and metabolization of new chemical entities. To date, most hPSC differentiation protocols yield hepatocyte-like cells with phenotypic characteristics of fetal rather than mature hepatocytes [4,9,10,14,18,24,37,38].

It is well known that regulation of gene expression is modulated by epigenetic modification caused by DNA methylation, histone modifications and miRNAs. DNA and chromatin modulation in both non-coding and coding regions, tightly regulate gene expression in hPSCs and their progeny. Polycomb complexes in part modulate chromatin structure during lineage commitment [39,40]. EZH2, a core component of PRC2, represses the expression of genes by functioning as a methyltransferase for H3K27me3 [41–43]. We previously published that persistent H3K27me3 marking of regulatory gene regions of hepatocyte genes is detected during hepatocyte differentiation from hPSCs, and this despite increased gene transcription. H3K27me3 marking might therefore not have a determinant role in gene activity in later stages of hepatocyte differentiation [24]. It might be possible that hPSC display a distinct histone modification state on regulatory elements at initial stages, a sort of chromatin “pre-pattern” that may reflect enhanced commitment to early-established lineage decisions under the direction of EZH2 expression.

To unravel to role of EZH2 during hepatocyte commitment, we assessed EZH2 transcript and protein expression during hepatocyte differentiation from hPSCs. EZH2 protein levels increased significantly on day 4 and day 8, but decreased to nearly zero on day 16 of differentiation. To determine if differentiation would be enhanced if levels of EZH2 were further induced during the initial stages of differentiation, we inducibly overexpressed *EZH2*, by incorporating a doxy inducible *EZH2* cassette by RMCE in the *AAVS1* locus of hPSCs created in our lab [26]. When *EZH2* expression was induced between day 0 and day 8 of the hepatocyte differentiation protocol, we demonstrated not only a more homogenous endoderm population on day 4, but also improved hepatoblast maturation on day 16. However, we demonstrated that doxy-mediated induction of *EZH2* is associated with a significant increase in overall H3K27me3 of hPSC progeny on day 4, day 8 and day 16. In line with our previous study, high levels of H3K27me3 staining persisted until day 16, and this despite the further commitment of endoderm cells to hepatoblasts [24]. Interestingly, we demonstrated that *ALBUMIN* and *AAT* hepatocyte marker genes showed less H3K27me3 at the end of the differentiation. We hypothesize that EZH2 overexpression might induce not- hepatocyte specific genes to retain H3K27me3 in favor of the expression of hepatocyte specific genes expression.

The decrease in *EZH2* expression from day 4 onwards despite continuous administration of doxy till day 8 was surprising. We previously described that inducible transgene expression from *AAVS1* was inhibited during hepatic differentiation through an unknown mechanism compatible with TRE-silencing triggered by TRE inactivity. However, inducible expression was robust through the whole differentiation process when doxy was applied starting from day-2 or day 4 [26]. As expression of EZH2 was induced on day 0, the *AAVS1*-mediated inhibition is highly unlikely to explain the down-regulation of EZH2 from day 4 onwards. This loss, therefore suggested post-transcriptional modulation of *EZH2* expression from day 4 onwards in the presence of doxycycline.

miRNAs are small non-coding RNAs that regulate the expression of more than 60% of protein coding genes in the human genome [44] by silencing target genes either via binding in a sequence specific manner messenger RNAs and cleaving them, or by inhibiting their translation [45]. miRNAs are known to regulate lineage-specific differentiation [46–51]. Expression of EZH2 can be modulated by direct binding of miRNAs, including miR-101, miR-138 and miR-214, to *EZH2* [33,34,52]. In line with this, we observed that that these miRNAs as well as an additional 8 miRNAs predicted to bind *EZH2*, were significantly induced from day 4 onwards, which coincides with the progressive decrease of *EZH2* mRNA and protein. Therefore, the loss of EZH2 from day 4 onwards despite transgenic overexpression until day 8, is likely due to miRNA mediated degradation, although further studies will be needed to demonstrate if miRNAs are the only responsible for this observation.

## Conclusion

In conclusion, we demonstrate that overexpression of *EZH2* early during the differentiation of hPSCs to hepatoblasts improved definitive endoderm formation and subsequent HLC generation. Surprisingly, despite doxy-mediated overexpression of *EZH2* until day 8 of differentiation, transcript and protein levels of EHZ2 decreased precipitously from day 4 onwards. This was concomitant with increased levels of miRNAs known/predicted to inhibit *EZH2* expression, which might be responsible for post-transcriptional regulation of EZH2 from day 4 onwards. In addition, despite the loss of *EZH2* expression, overall H3K27me3 levels remained high until day 16 of differentiation. In conclusion, we demonstrate that EZH2, of which the expression is tightly post-transcriptionally regulated, has a role in endoderm formation and enhanced levels of the Polycomb gene leads to improved hepatocyte differentiation.

## Supporting information

S1 Fig Polycomb group of genes expression. Relative gene expression (to *GAPDH*) of Polycomb group of genes (*EZH2*, *EZH1*, *SUZ12*, *EED*, *RING1B and BMI1*) in hPSCs, endoderm stage (day 4, endo), hepatoblast progenitor (day 8, HP) and fetal hepatocytes (day16, FH) of the hepatocytes differentiation. Data as mean ± SEM of n ≥ 3 IEs. * p < 0.05, ** p < 0.01.
(TIFF)

S2 Fig Characterization of hPS-*i*EZH2 cell line. **A.** Relative gene expression (to *GAPDH*) of pluripotent marker genes (*OCT4* and *NANOG)* in endo_d4 and HP_d8 untreated (-) and EZH2 doxy induced cells (+). Data as mean ± SEM. **B.** Relative gene expression of both endogenous and exogenous EZH2 (Total *EZH2*; * same analysis as in Fig 3A) and *EZH1* at endo_d4, HP_d8 and FH_d16 in untreated (-) and EZH2 doxy induced cells (+). Relative gene expression to GAPDH. Data as mean ± SEM. **C.** Relative gene expression of *RING1b* at endo_d4, HP_d8 and FH_d16 in untreated (-) and EZH2 doxy induced cells (+). Relative gene expression to GAPDH. Data as mean ± SEM.
(TIFF)

S3 Fig Hepatocytes markers expression. **A.** Relative gene expression (to *GAPDH*) of fetal (*CKIT*, *CYP3A7* and *CYP1A2*) and hepatic marker genes (*GSTA1*, *APOA1*, *NTCP*, *SRBI*, *MRP2*, *HNF3G*, *PEPCK*, *PXR*, *CYP2A6* and *CYP2C9*) and TFs (*CEBPB*, *P300* and *PROX1*) in HP_d8 and FH_d16 untreated (-) and EZH2 doxy induced cells (+). Data as mean ± SEM; protocol of differentiation and *EZH2* induction as in Fig 3A. **B.** Relative gene expression (to *GAPDH*) of pancreatic genes in HP_d8 and FH_d16 untreated (-) and EZH2 induced cells (+). Data as mean ± SEM.
(TIFF)

S4 Fig miRNAs analysis. **A.** The first 27 predicted miRNAs scored out of miRDB online database (http://mirdb.org/miRDB/) targeted EZH2 mRNA. **B.** Relative expression of 8 microRNAs (miR-31, miR-98, miR-125, miR-139, miR-181a, miR-181b, miR-200b and miR-217) during hepatocytes differentiation from hPSC-*i*EZH2 cell line doxy induced the first 8 days of differentiation. Relative gene expression to U6. Data as mean ± SEM of n = 3 IEs.
(TIFF)

## Abbreviations

hPSCs: Human Pluripotent Stem Cells
HLCs: Hepatocytes-Like Cells
RMCE: Recombinase-Mediated Cassette Exchange
GAPDH: GlycerAldehyde-3-Phosphate DeHydrogenase
DMSO: DiMethyl SulfOxide
TTS: Transcriptional Start Site
ORFs: Open Reading Frames
TFs: Transcription Factors
SEM: Standard Error of Mean
IEs: Independent Experiments

## References

[1] Bell CC, Hendriks DF, Moro SM, Ellis E, Walsh J, et al (2016) Characterization of primary human hepatocyte spheroids as a model system for drug-induced liver injury, liver function and disease. Sci Rep 6: 25187 doi: 10.1038/srep25187 27143246 10.1038/srep25187 PMC4855186

[2] Berger DR, Ware BR, Davidson MD, Allsup SR, Khetani SR (2015) Enhancing the functional maturity of induced pluripotent stem cell-derived human hepatocytes by controlled presentation of cell-cell interactions in vitro. Hepatology 61: 1370–1381. doi: 10.1002/hep.27621 25421237 10.1002/hep.27621

[3] Gieseck RL 3rd, Hannan NR, Bort R, Hanley NA, Drake RA, et al (2014) Maturation of induced pluripotent stem cell derived hepatocytes by 3D-culture. PLoS One 9: e86372 doi: 10.1371/journal.pone.0086372 24466060 10.1371/journal.pone.0086372 PMC3899231

[4] Hannan NR, Segeritz CP, Touboul T, Vallier L (2013) Production of hepatocyte-like cells from human pluripotent stem cells. Nat Protoc 8: 430–437. 23424751 10.1038/nprot.2012.153 PMC3673228

[5] Helsen N, Debing Y, Paeshuyse J, Dallmeier K, Boon R, et al (2016) Stem cell-derived hepatocytes: A novel model for hepatitis E virus replication. J Hepatol 64: 565–573. doi: 10.1016/j.jhep.2015.11.013 26626494 10.1016/j.jhep.2015.11.013

[6] Kondo Y, Ninomiya M, Kimura O, Machida K, Funayama R, et al (2014) HCV infection enhances Th17 commitment, which could affect the pathogenesis of autoimmune diseases. PLoS One 9: e98521 doi: 10.1371/journal.pone.0098521 24905921 10.1371/journal.pone.0098521 PMC4048196

[7] Roelandt P, Obeid S, Paeshuyse J, Vanhove J, Van Lommel A, et al (2012) Human pluripotent stem cell-derived hepatocytes support complete replication of hepatitis C virus. J Hepatol 57: 246–251. doi: 10.1016/j.jhep.2012.03.030 22521345 10.1016/j.jhep.2012.03.030

[8] Szkolnicka D, Zhou W, Lucendo-Villarin B, Hay DC (2013) Pluripotent stem cell-derived hepatocytes: potential and challenges in pharmacology. Annu Rev Pharmacol Toxicol 53: 147–159. doi: 10.1146/annurev-pharmtox-011112-140306 23294308 10.1146/annurev-pharmtox-011112-140306

[9] Baxter MA, Rowe C, Alder J, Harrison S, Hanley KP, et al (2010) Generating hepatic cell lineages from pluripotent stem cells for drug toxicity screening. Stem Cell Res 5: 4–22. doi: 10.1016/j.scr.2010.02.002 20483202 10.1016/j.scr.2010.02.002 PMC3556810

[10] Godoy P, Schmidt-Heck W, Natarajan K, Lucendo-Villarin B, Szkolnicka D, et al (2015) Gene networks and transcription factor motifs defining the differentiation of stem cells into hepatocyte-like cells. J Hepatol 63: 934–942. doi: 10.1016/j.jhep.2015.05.013 26022688 10.1016/j.jhep.2015.05.013 PMC4580233

[11] Godoy P, Widera A, Schmidt-Heck W, Campos G, Meyer C, et al (2016) Gene network activity in cultivated primary hepatocytes is highly similar to diseased mammalian liver tissue. Arch Toxicol 90: 2513–2529. doi: 10.1007/s00204-016-1761-4 27339419 10.1007/s00204-016-1761-4 PMC5043005

[12] Heslop JA, Rowe C, Walsh J, Sison-Young R, Jenkins R, et al (2016) Mechanistic evaluation of primary human hepatocyte culture using global proteomic analysis reveals a selective dedifferentiation profile. Arch Toxicol. 10.1007/s00204-016-1694-y PMC5225178 27039104

[13] Mann DA (2015) Human induced pluripotent stem cell-derived hepatocytes for toxicology testing. Expert Opin Drug Metab Toxicol 11: 1–5. doi: 10.1517/17425255.2015.981523 25385341 10.1517/17425255.2015.981523

[14] Ulvestad M, Nordell P, Asplund A, Rehnstrom M, Jacobsson S, et al (2013) Drug metabolizing enzyme and transporter protein profiles of hepatocytes derived from human embryonic and induced pluripotent stem cells. Biochem Pharmacol 86: 691–702. doi: 10.1016/j.bcp.2013.06.029 23856292 10.1016/j.bcp.2013.06.029

[15] Cao R, Wang L, Wang H, Xia L, Erdjument-Bromage H, et al (2002) Role of histone H3 lysine 27 methylation in Polycomb-group silencing. Science 298: 1039–1043. doi: 10.1126/science.1076997 12351676 10.1126/science.1076997

[16] Margueron R, Reinberg D (2011) The Polycomb complex PRC2 and its mark in life. Nature 469: 343–349. doi: 10.1038/nature09784 21248841 10.1038/nature09784 PMC3760771

[17] Sauvageau M, Sauvageau G (2010) Polycomb group proteins: multi-faceted regulators of somatic stem cells and cancer. Cell Stem Cell 7: 299–313. doi: 10.1016/j.stem.2010.08.002 20804967 10.1016/j.stem.2010.08.002 PMC4959883

[18] Chen YH, Hung MC, Li LY (2012) EZH2: a pivotal regulator in controlling cell differentiation. Am J Transl Res 4: 364–375. 23145205 PMC3493026

[19] Deb G, Singh AK, Gupta S (2014) EZH2: not EZHY (easy) to deal. Mol Cancer Res 12: 639–653. doi: 10.1158/1541-7786.MCR-13-0546 24526064 10.1158/1541-7786.MCR-13-0546 PMC4020974

[20] Collinson A, Collier AJ, Morgan NP, Sienerth AR, Chandra T, et al (2016) Deletion of the Polycomb-Group Protein EZH2 Leads to Compromised Self-Renewal and Differentiation Defects in Human Embryonic Stem Cells. Cell Rep 17: 2700–2714. doi: 10.1016/j.celrep.2016.11.032 27926872 10.1016/j.celrep.2016.11.032 PMC5177603

[21] Chamberlain SJ, Yee D, Magnuson T (2008) Polycomb repressive complex 2 is dispensable for maintenance of embryonic stem cell pluripotency. Stem Cells 26: 1496–1505. doi: 10.1634/stemcells.2008-0102 18403752 10.1634/stemcells.2008-0102 PMC2630378

[22] Pasini D, Bracken AP, Hansen JB, Capillo M, Helin K (2007) The polycomb group protein Suz12 is required for embryonic stem cell differentiation. Mol Cell Biol 27: 3769–3779. doi: 10.1128/MCB.01432-06 17339329 10.1128/MCB.01432-06 PMC1899991

[23] Aoki R, Chiba T, Miyagi S, Negishi M, Konuma T, et al (2010) The polycomb group gene product Ezh2 regulates proliferation and differentiation of murine hepatic stem/progenitor cells. J Hepatol 52: 854–863. doi: 10.1016/j.jhep.2010.01.027 20395008 10.1016/j.jhep.2010.01.027

[24] Vanhove J, Pistoni M, Welters M, Eggermont K, Vanslembrouck V, et al (2016) H3K27me3 Does Not Orchestrate the Expression of Lineage-Specific Markers in hESC-Derived Hepatocytes In Vitro. Stem Cell Reports 7: 192–206. doi: 10.1016/j.stemcr.2016.06.013 27477635 10.1016/j.stemcr.2016.06.013 PMC4982990

[25] Thomson JA, Itskovitz-Eldor J, Shapiro SS, Waknitz MA, Swiergiel JJ, et al (1998) Embryonic stem cell lines derived from human blastocysts. Science 282: 1145–1147. 9804556 10.1126/science.282.5391.1145

[26] Ordovas L, Boon R, Pistoni M, Chen Y, Wolfs E, et al (2015) Efficient Recombinase-Mediated Cassette Exchange in hPSCs to Study the Hepatocyte Lineage Reveals AAVS1 Locus-Mediated Transgene Inhibition. Stem Cell Reports 5: 918–931. doi: 10.1016/j.stemcr.2015.09.004 26455413 10.1016/j.stemcr.2015.09.004 PMC4649136

[27] Pistoni M, Verrecchia A, Doni M, Guccione E, Amati B (2010) Chromatin association and regulation of rDNA transcription by the Ras-family protein RasL11a. EMBO J 29: 1215–1224. doi: 10.1038/emboj.2010.16 20168301 10.1038/emboj.2010.16 PMC2857460

[28] Izzi B, Binder AM, Michels KB (2014) Pyrosequencing Evaluation of Widely Available Bisulfite Conversion Methods: Considerations for Application. Med Epigenet 2: 28–36. doi: 10.1159/000358882 24944560 10.1159/000358882 PMC4058339

[29] Xu CR, Cole PA, Meyers DJ, Kormish J, Dent S, et al (2011) Chromatin "prepattern" and histone modifiers in a fate choice for liver and pancreas. Science 332: 963–966. doi: 10.1126/science.1202845 21596989 10.1126/science.1202845 PMC3128430

[30] Xu CR, Li LC, Donahue G, Ying L, Zhang YW, et al (2014) Dynamics of genomic H3K27me3 domains and role of EZH2 during pancreatic endocrine specification. EMBO J 33: 2157–2170. doi: 10.15252/embj.201488671 25107471 10.15252/embj.201488671 PMC4282504

[31] Friedman JM, Liang G, Liu CC, Wolff EM, Tsai YC, et al (2009) The putative tumor suppressor microRNA-101 modulates the cancer epigenome by repressing the polycomb group protein EZH2. Cancer Res 69: 2623–2629. doi: 10.1158/0008-5472.CAN-08-3114 19258506 10.1158/0008-5472.CAN-08-3114

[32] Varambally S, Cao Q, Mani RS, Shankar S, Wang X, et al (2008) Genomic loss of microRNA-101 leads to overexpression of histone methyltransferase EZH2 in cancer. Science 322: 1695–1699. doi: 10.1126/science.1165395 19008416 10.1126/science.1165395 PMC2684823

[33] Zhang H, Zhang H, Zhao M, Lv Z, Zhang X, et al (2013) MiR-138 inhibits tumor growth through repression of EZH2 in non-small cell lung cancer. Cell Physiol Biochem 31: 56–65. doi: 10.1159/000343349 23343715 10.1159/000343349

[34] Juan AH, Kumar RM, Marx JG, Young RA, Sartorelli V (2009) Mir-214-dependent regulation of the polycomb protein Ezh2 in skeletal muscle and embryonic stem cells. Mol Cell 36: 61–74. doi: 10.1016/j.molcel.2009.08.008 19818710 10.1016/j.molcel.2009.08.008 PMC2761245

[35] Cao Q, Mani RS, Ateeq B, Dhanasekaran SM, Asangani IA, et al (2011) Coordinated regulation of polycomb group complexes through microRNAs in cancer. Cancer Cell 20: 187–199. doi: 10.1016/j.ccr.2011.06.016 21840484 10.1016/j.ccr.2011.06.016 PMC3157014

[36] Leung-Kuen AS, Oi-Lin NI, Chun-Ming W (2013) Epigenetic Regulation of EZH2 and Its Targeted miRNAs eBook—Epigenetic and Cancer.

[37] Shan J, Schwartz RE, Ross NT, Logan DJ, Thomas D, et al (2013) Identification of small molecules for human hepatocyte expansion and iPS differentiation. Nat Chem Biol 9: 514–520. doi: 10.1038/nchembio.1270 23728495 10.1038/nchembio.1270 PMC3720805

[38] Siller R, Greenhough S, Naumovska E, Sullivan GJ (2015) Small-molecule-driven hepatocyte differentiation of human pluripotent stem cells. Stem Cell Reports 4: 939–952. doi: 10.1016/j.stemcr.2015.04.001 25937370 10.1016/j.stemcr.2015.04.001 PMC4437467

[39] Lee TI, Jenner RG, Boyer LA, Guenther MG, Levine SS, et al (2006) Control of developmental regulators by Polycomb in human embryonic stem cells. Cell 125: 301–313. doi: 10.1016/j.cell.2006.02.043 16630818 10.1016/j.cell.2006.02.043 PMC3773330

[40] Surface LE, Thornton SR, Boyer LA (2010) Polycomb group proteins set the stage for early lineage commitment. Cell Stem Cell 7: 288–298. doi: 10.1016/j.stem.2010.08.004 20804966 10.1016/j.stem.2010.08.004

[41] Christophersen NS, Helin K (2010) Epigenetic control of embryonic stem cell fate. J Exp Med 207: 2287–2295. doi: 10.1084/jem.20101438 20975044 10.1084/jem.20101438 PMC2964577

[42] Ezhkova E, Lien WH, Stokes N, Pasolli HA, Silva JM, et al (2011) EZH1 and EZH2 cogovern histone H3K27 trimethylation and are essential for hair follicle homeostasis and wound repair. Genes Dev 25: 485–498. doi: 10.1101/gad.2019811 21317239 10.1101/gad.2019811 PMC3049289

[43] Hansen KH, Bracken AP, Pasini D, Dietrich N, Gehani SS, et al (2008) A model for transmission of the H3K27me3 epigenetic mark. Nat Cell Biol 10: 1291–1300. doi: 10.1038/ncb1787 18931660 10.1038/ncb1787

[44] Friedman RC, Farh KK, Burge CB, Bartel DP (2009) Most mammalian mRNAs are conserved targets of microRNAs. Genome Res 19: 92–105. doi: 10.1101/gr.082701.108 18955434 10.1101/gr.082701.108 PMC2612969

[45] Hutvagner G, Zamore PD (2002) A microRNA in a multiple-turnover RNAi enzyme complex. Science 297: 2056–2060. doi: 10.1126/science.1073827 12154197 10.1126/science.1073827

[46] Alvarez-Garcia I, Miska EA (2005) MicroRNA functions in animal development and human disease. Development 132: 4653–4662. doi: 10.1242/dev.02073 16224045 10.1242/dev.02073

[47] Chen Y, Verfaillie CM (2014) MicroRNAs: the fine modulators of liver development and function. Liver Int 34: 976–990. doi: 10.1111/liv.12496 24517588 10.1111/liv.12496

[48] Delaloy C, Liu L, Lee JA, Su H, Shen F, et al (2010) MicroRNA-9 coordinates proliferation and migration of human embryonic stem cell-derived neural progenitors. Cell Stem Cell 6: 323–335. doi: 10.1016/j.stem.2010.02.015 20362537 10.1016/j.stem.2010.02.015 PMC2851637

[49] Ivey KN, Srivastava D (2010) MicroRNAs as regulators of differentiation and cell fate decisions. Cell Stem Cell 7: 36–41. doi: 10.1016/j.stem.2010.06.012 20621048 10.1016/j.stem.2010.06.012

[50] Kim N, Kim H, Jung I, Kim Y, Kim D, et al (2011) Expression profiles of miRNAs in human embryonic stem cells during hepatocyte differentiation. Hepatol Res 41: 170–183. doi: 10.1111/j.1872-034X.2010.00752.x 21269386 10.1111/j.1872-034X.2010.00752.x

[51] Pobezinsky LA, Etzensperger R, Jeurling S, Alag A, Kadakia T, et al (2015) Let-7 microRNAs target the lineage-specific transcription factor PLZF to regulate terminal NKT cell differentiation and effector function. Nat Immunol 16: 517–524. doi: 10.1038/ni.3146 25848867 10.1038/ni.3146 PMC4406853

[52] Benetatos L, Voulgaris E, Vartholomatos G, Hatzimichael E (2013) Non-coding RNAs and EZH2 interactions in cancer: long and short tales from the transcriptome. Int J Cancer 133: 267–274. doi: 10.1002/ijc.27859 23001607 10.1002/ijc.27859

