# Supplementary figures and images for: Dynamic regulation of EZH2 from HPSc to hepatocyte-like cell fate
Source: PLoS One. 2017 Nov 1;12(11):e0186884. doi: 10.1371/journal.pone.0186884 (PMC5665677; doi:10.1371/journal.pone.0186884)

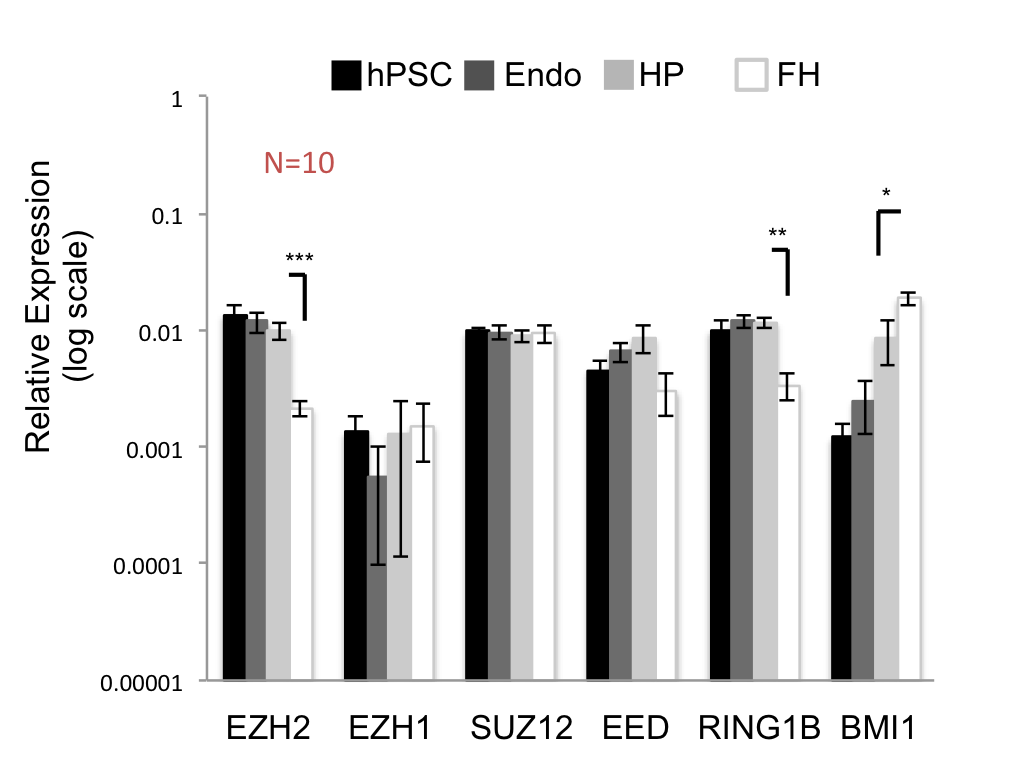

Supplement: S1 Fig — Relative gene expression (to GAPDH) of Polycomb group of genes (EZH2, EZH1, SUZ12, EED, RING1B and BMI1) in hPSCs, endoderm stage (day 4, endo), hepatoblast progenitor (day 8, HP) and fetal hepatocytes (day16, FH) of the hepatocytes differentiation. Data as mean ± SEM of n ≥ 3 IEs. * p < 0.05, ** p < 0.01. (TIFF) [file pone.0186884.s001.tiff]

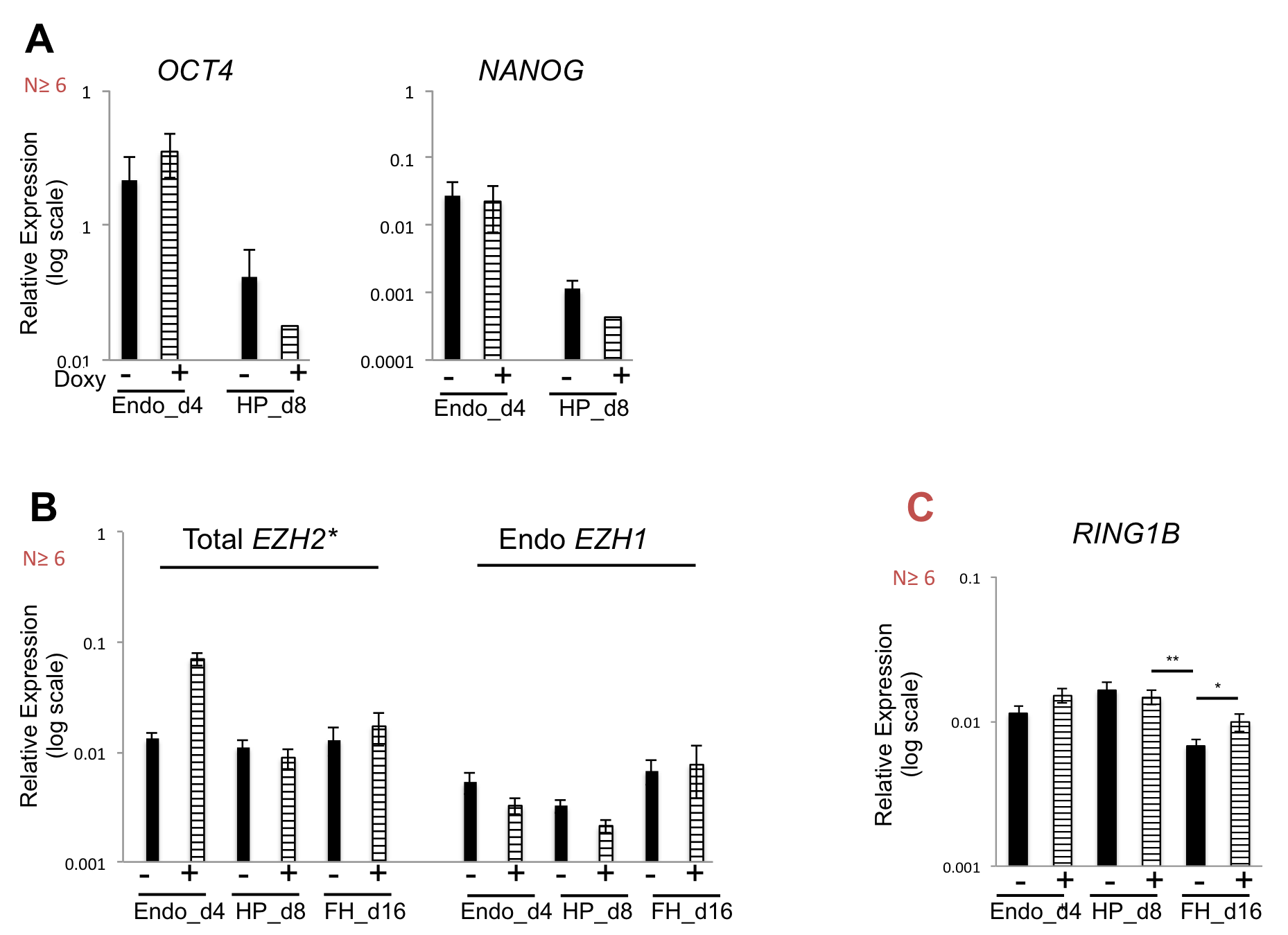

Supplement: S2 Fig — A. Relative gene expression (to GAPDH) of pluripotent marker genes (OCT4 and NANOG) in endo_d4 and HP_d8 untreated (-) and EZH2 doxy induced cells (+). Data as mean ± SEM. B. Relative gene expression of both endogenous and exogenous EZH2 (Total EZH2; * same analysis as in Fig 3A) and EZH1 at endo_d4, HP_d8 and FH_d16 in untreated (-) and EZH2 doxy induced cells (+). Relative gene expression to GAPDH. Data as mean ± SEM. C. Relative gene expression of RING1b at endo_d4, HP_d8 and FH_d16 in untreated (-) and EZH2 doxy induced cells (+). Relative gene expression to GAPDH. Data as mean ± SEM. (TIFF) [file pone.0186884.s002.tiff]

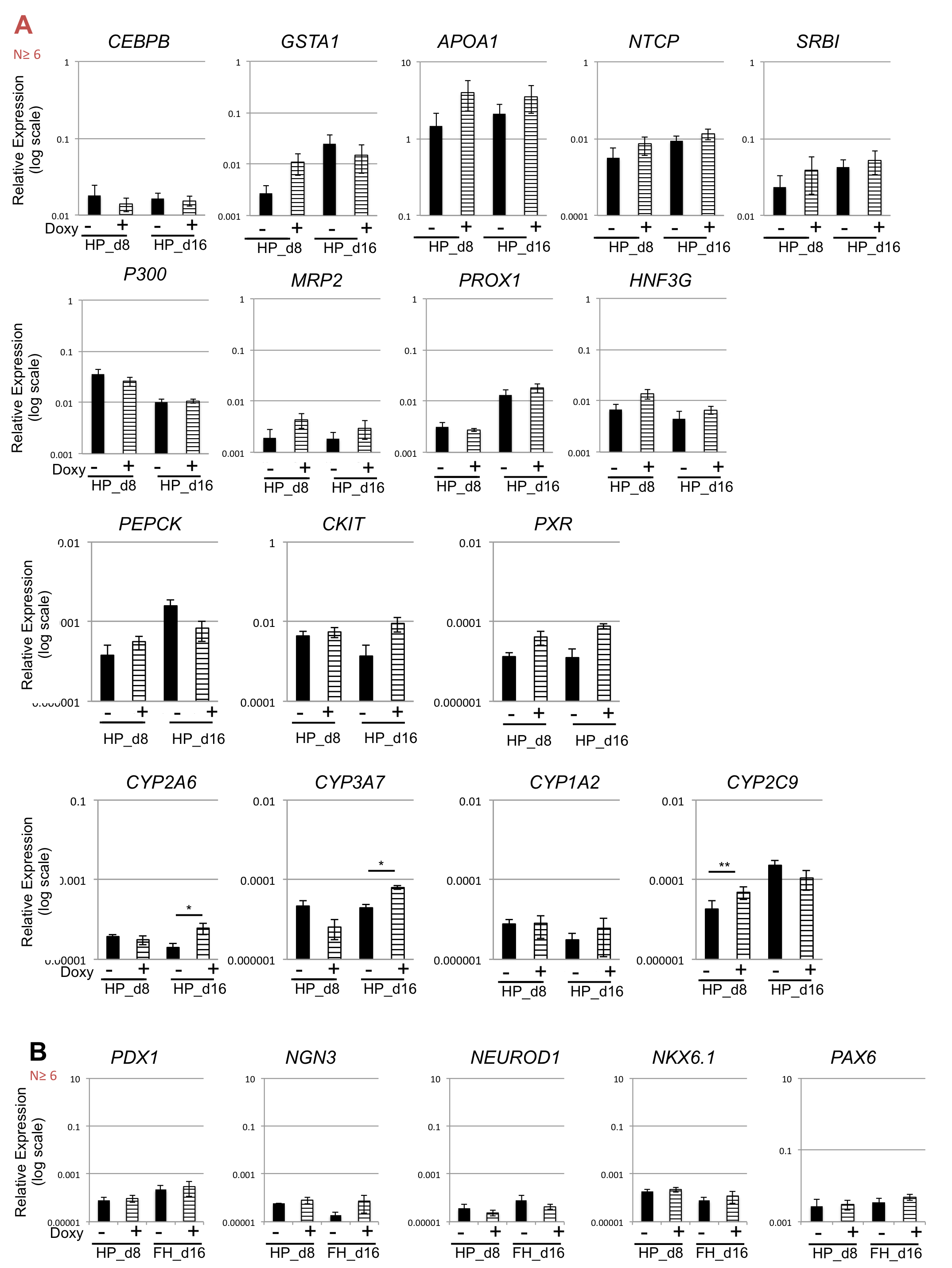

Supplement: S3 Fig — A. Relative gene expression (to GAPDH) of fetal (CKIT, CYP3A7 and CYP1A2) and hepatic marker genes (GSTA1, APOA1, NTCP, SRBI, MRP2, HNF3G, PEPCK, PXR, CYP2A6 and CYP2C9) and TFs (CEBPB, P300 and PROX1) in HP_d8 and FH_d16 untreated (-) and EZH2 doxy induced cells (+). Data as mean ± SEM; protocol of differentiation and EZH2 induction as in Fig 3A. B. Relative gene expression (to GAPDH) of pancreatic genes in HP_d8 and FH_d16 untreated (-) and EZH2 induced cells (+). Data as mean ± SEM. (TIFF) [file pone.0186884.s003.tiff]

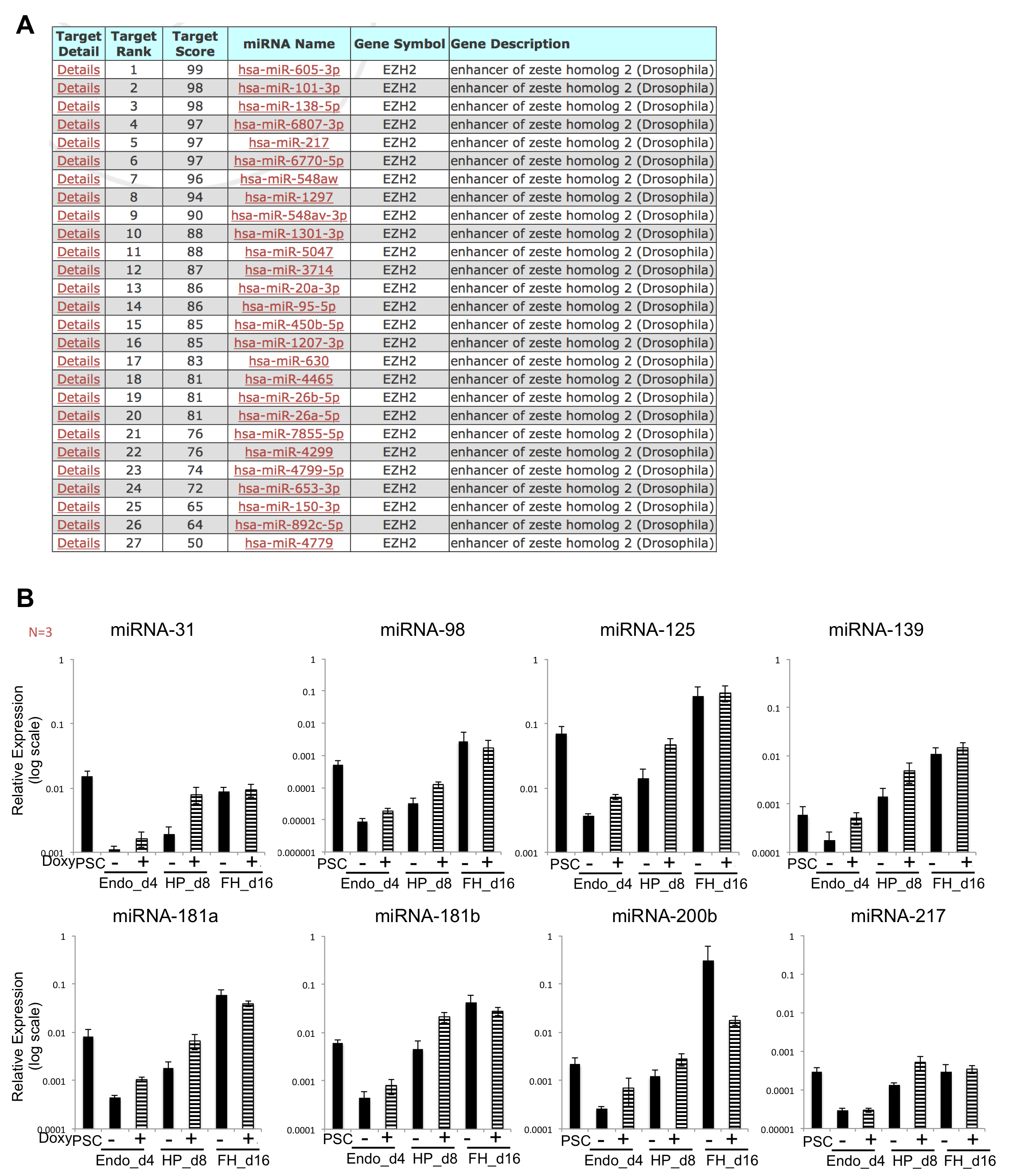

Supplement: S4 Fig — A. The first 27 predicted miRNAs scored out of miRDB online database (http://mirdb.org/miRDB/) targeted EZH2 mRNA. B. Relative expression of 8 microRNAs (miR-31, miR-98, miR-125, miR-139, miR-181a, miR-181b, miR-200b and miR-217) during hepatocytes differentiation from hPSC-iEZH2 cell line doxy induced the first 8 days of differentiation. Relative gene expression to U6. Data as mean ± SEM of n = 3 IEs. (TIFF) [file pone.0186884.s004.tiff]
